# Recent Changes in Glacial Area and Volume on Tuanjiefeng Peak Region of Qilian Mountains, China

**DOI:** 10.1371/journal.pone.0070574

**Published:** 2013-08-27

**Authors:** Junli Xu, Shiyin Liu, Shiqiang Zhang, Wanqin Guo, Jian Wang

**Affiliations:** 1 State Key Laboratory of Cryosphere Science, Cold and Arid Regions Environmental and Engineering Research Institute, Chinese Academy of Sciences, Lanzhou, PR China; 2 The Land in the Earth System, Max Planck Institute for Meteorology, Hamburg, Germany; The Ohio State University, United States of America

## Abstract

Glaciers' runoff in the Qilian Mountains serves as a critical water resource in the northern sections of the Gansu province, the northeastern sections of the Qinghai province, and the northeastern fringe of the Tibetan Plateau. Changes in the glacial area and volume around the highest peak of the Qilian Mountains, i.e., Tuanjiefeng Peak, were estimated using multi-temporal remote-sensing images and digital elevation models, and all possible sources of uncertainty were considered in detail. The total glacier area decreased by 16.1±6.34 km^2^ (9.9±3.9%) during 1966 to 2010. The average annual glacier shrinkage was −0.15% a^−1^ from 1966 to 1995, −0.61% a^−1^ from 1995 to 2000, −0.20% a^−1^ from 2000 to 2006, and −0.45% a^−1^ from 2006 to 2010. A comparison of glacier surface elevations using digital elevation models derived from topographic maps in 1966 and from the Shuttle Radar Topography Mission in 1999 suggests that 65% of the grid cells has decreased, thereby indicating that the glacier thickness has declined. The average change in glacier thickness was −7.3±1.5 m (−0.21±0.04 m·a^−1^) from 1966 to 1999. Glaciers with northeastern aspects thinned by 8.3±1.4 m from 1966 to 1999, i.e., almost twice as much as those with southwestern aspects (4.3±1.3 m). The ice volume decreased by 11.72±2.38×10^8^ m^3^ from 1966 to 1999, which was about 17.4% more than the value calculated from the statistical relationship between glacier area and volume. The relationship between glacier area change and elevation zone indicates that glacier change is not only dominated by climate change but also affected by glacier dynamics, which are related to local topography. The varied response of a single glacier to climate change indicates that the glacier area change scheme used in some models must be improved.

## Introduction

Glaciers can serve as a sensitive barometer of climate change and play an important role in the climate system by growing or shrinking in response to changes in temperature and snowfall [1]. Glaciers are also important fresh water resources in many arid and semi-arid regions, like western China [2]. In recent decades, changes in the lengths and areas of glaciers have been documented through geodetic surveying, geographic information systems, and remote-sensing data. Most research has indicated that glaciers are rapidly retreating in terms of length and shrinking in terms of area in most glaciated areas of western China [3]–[6] and other regions of the world [7]–[9]. Consequently, glacier runoff is expected to have changed as well [10]–[12].

Changes in ice thickness and volume are important and direct evidence of glacial changes in studies on basin water resources. The traditional methods of measuring changes in ice thickness is to repeatedly measure glacier thickness using ground penetrating radar or repeatedly measure glacier mass balance via snow pits. The impact of regional and global glacier changes has been estimated based on the extrapolation of measurements of mass balance for limited benchmarks glaciers in a particular region [13], [14]; however, the number of benchmark glaciers in china is very limited. About 34 glaciers in China have been measured for several months, i.e., up to one or two years. Only the Meikuang Glacier, the Dongkemadi Glacier and Glacier No. 1 at the headwaters of the Urümqi River have been measured consecutively for more than 10 years [15].

Changes in ice thickness also can be derived from difference observed in glacier surface elevation at different time periods in the field survey. This has been accomplished through repeated measuring by real-time kinematic global positioning system surveys in western China [6], [16], [17], but these surveys are difficult to carry out for a large number of glaciers due to the high costs of the surveys and the hostile environment on the glacier. An alternative method of measuring the changes in glacier surface elevation involves the use of multiple temporal digital elevation models (DEMs). Indeed, DEMs generated from topographical maps and/or aerial photographs have often been used as reference data [18]–[20]. Recently, the Shuttle Radar Topography Mission (SRTM), and the Geoscience Laser Altimeter System which carried onboard the Ice, Cloud, and Land Elevation Satellite (ICESat), were used in studies on glaciers changes in Antarctica, Greenland, Alaska Svalbard, the Alps, and the central Tien Shan Mountains [20]–[25]. Comparisons between DEMs suggest that glacier surface elevation changes varied greatly among the different regions, and more data and results are needed in order to understand glacier response to global warming.

Glaciers runoff in the Qilian Mountains is a critical water resource for northern sections of the Gansu province and northeastern sections of the Qinghai province. Glaciers around Tuanjiefeng (TJF, also known as Kangze'gyai), i.e., the highest peak in the Qilian Mountains, are the main water resource for the rivers in the Qilian mountains. The glaciers in the area of the western Qilian Mountains, including TJF, have receded by about 16.9% from the end of Little Ice Age (LIA, from 1622 to 1740) to 1990 [26]. The glacier area on the Su-lo Mountains, part of western Qilian Mountains, including TJF, reported to decrease from 429.1 km^2^ in 1966 to 458.2 km^2^ in 1999, and the loss in the ice volume were estimated at 1.4 km^3^ from 1966 to 2000 via a comparison of the glacier surface elevation between SRTM and DEM in 1966 [27]. However, this study did not account for the error associated with the shift between DEMs and the different resolution [28], [29].

On the other hand, recent research has suggested that glacier shrinkage began accelerating in 2000 in the central Qilian Mountains [17], [30]. Thus, this study was designed to investigate the glacier length and area changes in TJF during 1966 to 2010 via topographic maps from 1966 and Landsat Multispectral Scanner (MSS)/Thematic Mapper (TM)/Enhanced Thematic Mapper Plus (ETM+) imagery from 1973, 1995, 1999, 2006, and 2010; moreover, the ice surface elevation and volume changes in the glaciers around TJF from 1966 to 1999 were investigated by comparing SRTM DEM and the DEM from 1966, and the accuracy of the results were considered in detail.

## Study Area

**Figure 1:**
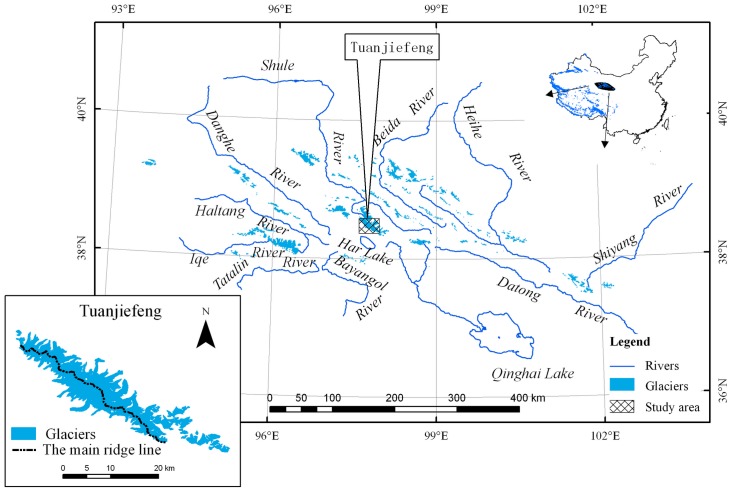
Location of the Tuanjiefeng region in the Qilian Mountains on the northeastern Tibet Plateau, China. The lower left map is the distribution of glaciers in the region.

The TJF region (36.5°–39°N, 93.5°–103°E) is located on the northeastern fringe of the Tibetan Plateau (Figure 1). The peak of the TJF region is 5826 m above Sea level (ASL), and the average firn line is at about 4862 m ASL during late summer [31]. The average annual precipitation and temperature at 4000 m ASL are about 300 mm and −6.3°C, respectively [31]. According to the China Glacier Inventory [32], 98 glaciers with a total area of 162.7 km^2^ can be found in the TJF region. Most of the glaciers in this area (i.e., 90.8%) are smaller than 5 km^2^, and the combined area of these smaller glaciers account for 54.2% of the total glacier area. There are only nine glaciers larger than 5 km^2^. Glaciers around TJF are separated by a main ridge line from northwest to southeast, thus, all the glaciers have southwestern or northeastern aspects. Note that glaciers with a northeasterly aspect cover an area of 105.57 km^2^ (i.e., 64.9% of the total area for all glaciers in the region). All glaciers in the TJF region are free of debris.

## Datasets

### SRTM

SRTM imagery was used to divide glacier basins into individual glaciers and to study ice surface elevation changes. The SRTM has a specially modified radar system that was used onboard the space shuttle *Endeavour* during an 11-day mission in February of 2000. SRTM elevation data are available between the latitudes of 60°N and 57°S. The geodetic reference for SRTM data is the World Geodetic System 1984 (WGS84), defined as the 1996 Earth Gravitational Model (EGM96) geoid.

The horizontal resolution of the SRTM DEM is 90 m, and the absolute vertical accuracy is better than 9 m [33]. A previous study [34] found that the average elevation difference between the SRTM DEM and the topographic map was about 6 m in the Tien Shan Mountains.

The penetrating depth of the C band radar on snow is highly dependent on the wetness of the snow. Wet snow will show no significant penetration. It increases from 0 to 4 m for Alaskan glaciers and up to 10 m on the dry, cold firn of the Greenland ice sheet [35].

The climate for an extensive region of Asia involves highly concentrated summer precipitation climate [36]. In fact, the total winter precipitation (i.e., for December of 1999 and January and February of 2000) was only 4.5 mm, which accounted for 1.2% of the annual precipitation in 2000 in the TJF region. Therefore, the measurable accumulated snow in February of 2000 was very small. On the other hand, the average air temperature during the Winter of 2000 was about −15.9°C in the TJF region, and the average air temperature over the glacier was lower than −15.9°C due to a lapse rate of about 0.6°C/100 m, which indicates that the small amount of accumulated snow nearly had no chance to melt in the Winter of 2000 in the TJF region. Therefore, the snow should be relatively dry, thereby allowing the C band radar to penetrate into dry snow and perhaps even reach 10 meters of depth. Thus, the elevation of the SRTM data referred to the 1999 glacier surface at the end of the melt season [28], [37].

SRTM DEM version filled finished_B was downloaded from the Global Land Cover Facility (http://glcf.umd.edu/data/srtm/). This DEM data was adjusted for voids of high quality and to address minimal gaps in the data. The quality of the SRTM DEM in the TJF region was also checked via visual examination.

We tried to find other stereo image pairs in the TJF region to construct the DEM ourselves or other DEM products after 1999, but no qualified satellite images or products available due to snow cover and cloudy cover.

### DEM 1966

Four 1∶50,000 topographic maps, which were compiled from 1∶40,000 aerial stereo pairs taken in 1966 by the State Bureau of Surveying and Mapping of China (SBSMC), were collected and merged into one DEM called DEM 1966. The geographic projection of the topographic maps was based on the Beijing Geodetic Coordinate System 1954 (BJ54) geoid and the Yellow Sea 1956 datum (i.e., the mean sea level of the Yellow Sea at Qingdao Tidal Observatory in 1956).

The contour lines of digital elevation was obtained from SBSMC and were digitized from topographic maps and re-projected as the Xian Geodetic Coordinate System 1980 (GDZ80) geoid using the Yellow Sea 1985 datum. The errors in the 1966 contours were reported as 3–5 m over flat and hilly areas and 8–14 m over mountains [38]. Note that the digital elevation of the contours on the snow probably contains larger errors than digital elevations of areas on an ice-free mountain due to the lack of visible contrast on the surface of the snow. The data were mainly collected in the late summer or early autumn, i.e., when most of the snow has melted. On the other hand, a large scale field survey was conducted in which part of the survey took place on glacier areas with snow covered surfaces, and some experts provided visual interpretation for the snow covered areas. Because we did not have enough original field survey data in 1966 around the TJF, this error is difficult to measure and was ignored in this study.

The contour lines of DEM 1966 were re-projected to UTM zone 47N using a seven parameter transformation method that will be described subsequently and interpolated to 30 m resolution by employing the Thiessen polygon method. More than 12 triangulation points around the TJF, the GDZ80 geoid and WGS84 ellipsoid elevations obtained from the National Geomatics Center of China were used for seven parameter transformation.

### Landsat satellite imagery

All Landsat images, i.e., the MSS, TM, and ETM+ images, were used in this study (Table 1). Landsat MSS images have a spatial resolution of 57 m and five spectral bands (i.e., two visible bands, two near-infrared bands, and one thermal band). The TM images have a spatial resolution of 30 m, and include seven spectral bands (i.e., blue, green, and red visible bands, one near-infrared band, two short-infrared bands, and one thermal band). The ETM+ images have a panchromatic band with a spatial resolution of 15 m plus the TM bands. Because of the low resolution and snow cover, Landsat MSS data were not used for g the whole area; instead, only the data for four typical glaciers were used. The orthorectified Landsat MSS 1973 and TM 1995 (L1G) were collected from the Global Land Cover Facility (GLCF: http://www.landcover.org). The other orthorectified images (L1T) were obtained from the United States Geological Survey (USGS: http://landsat.usgs.gov). For all of the Landsat images, UTM zone 47N projection was used.

**Table 1:** Metadata of Landsat satellite images around the Tuanjiefeng region, Qilian Mountain, China.

Sensor	Date (mm-dd-yyyy)	Path/row	Resolution/m
MSS	10-28-1973	145/33	57
TM	8-19-1995	135/33	30
ETM+	16-8-1999	135/33	15
TM	8-1-2006	135/33	30
TM	9-13-2010	135/33	30

## Methods

### Glacier boundary extraction

Four topographic maps of the TJF scanned in 600 DPI resolution were geo-registered, and the Beijing 1954 GK zone 17N projection was used. The root mean square error (RMSE) of the geo-registration was 18.6 m. Glacier outlines from 1966 were digitized on these maps using the ArcGIS 9.3 software and were transformed to UTM zone 47N projection based on a seven parameter transformation model.

Several glacier boundaries for 1973 were digitized using the Landsat MSS false-color composite 4, 3, 2 (i.e., R, G, B, respectively) imagery. For 1995, 1999, 2006, and 2010, the glacier outlines were obtained via the automatic delineation of a Landsat TM/ETM+ band ratio image of TM3/TM5 with a threshold of 2.1. This approach has been successfully applied in other studies to extract the outlines of many glaciated areas [39]. We use the method [7] to divide glacier boundaries into individual glaciers. All of the boundaries of each glacier were visually checked after extraction.

### Uncertainty in extracting glacier boundaries

The best way to assess the accuracy of glacier boundary extraction via Landsat TM/ETM+ images is to compare the extracted boundaries with a higher resolution satellite image [40]. However, obtaining high resolution images during similar time periods as the Landsat TM/ETM+ images was difficult. We attempted to assess the uncertainty associated with the glacier boundaries for each period by looking at the influencing factors (e.g., image resolution), errors in co-registration, and scene quality (e.g., clouds, seasonal snow, and shadow).

Because the accurate detection glacier changes that are smaller than the resolution of the measurement is impossible, we assumed that all of the outermost pixels over the glacier in question were uncertain. Therefore, the number of uncertain grids was assumed to be the number of grids passing through the glacier boundary perimeter. In general, three kinds of glacier boundaries are extracted from raster to vector, i.e., the stair-shape, the straight line, and the tooth-shape. However, most boundaries are stair-shaped, and only a few boundaries are like the latter two. The tooth-shape is usually formed in some sharp terminals of glaciers, and the influence of these sharp terminals on the number of pixels can be omitted. The straight line is found in a few glaciers. Therefore, the number of uncertain grids can be estimated by the following equation:
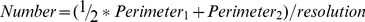
(1)

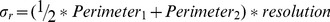
(2)where *Perimeter_1_* is the total length of the stair-shapes boundaries, *Perimeter_2_* is the total length of the straight line boundaries, and the resolution is the image resolution. Thus, the length uncertainty is the one pixel of images, and the area uncertainty σ_r_ is the total area of the “number” glaciers.

To estimate errors in the co-registration of topographic maps as compared to that of the Landsat images, 20 independent tie points were selected. The co-registration accuracy of between topographic map and MSS 1973, TM 1995, ETM+ 1999, TM 2006 and TM 2010 was 30.6 m, 15.4 m, 11.8 m, 16.9 m, and 14.7 m, respectively. In all cases, the error was less than the resolution of the image (i.e., one pixel), and the error was assosiated with the uncertainty of the outermost glacier pixels.

The influence of cloud and shadow was considered negligible. No seasonal snow was involved except with the Landsat MSS images. The MSS generated a 1% difference in snow cover as compared with the other TM images. This error was added to the overall error.

The total error is calculated using the flowing equation
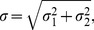
(3)where *σ* is the total error, *σ_1_* is the error from the first image and *σ_2_* is the error from the second image.

### Seven parameter transformation

Shifts and deformations occur in three-dimensional directions between the coordinates of the DEM 1966 and the SRTM DEM due to the different geoid and datum used in each of them. A seven parameter transformation (i.e., so named for the three translations, one scaling operation, and three rotations) called the Helmert transformation was used to transform the Xian 80 data into the WGS84 data in this study. The datum transformation model is written as:
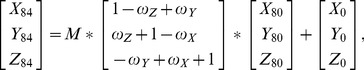
(4)where *X_84_*, *Y_84_* and *Z_84_* are the WGS84 datum coordinates, *X_80_*, *Y_80_* and *Z_80_* are the Xian 80 datum coordinates, *X_0_*, *Y_0_*, and *Z_0_* are the translation parameters, 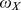, 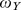, and 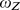 are the rotation parameters; and M is the scale factor. These parameters for each map were estimated from three known national trigonometric points. The mean error is 0.5 m in the vertical direction when validated by 8 kinematic Global Positioning System points. The accuracy of this seven parameter transformation has also proved to be less than 0.5 m by other studies [4], [41].

### DEMs accuracy and change correction

The surface elevation accuracy of SRTM DEM and DEM 1966 for the TJF was tested by comparing the over barren areas with the GLA14 product of the National Aeronautics and Space Administration's (NASA) Ice, Cloud, and Land Elevation Satellite (ICESat) Geoscience Laser Altimeter System (GLAS) (http://nsidc.org/data/icesat/). Four hundred and thirty GLAS footprints over barren areas in 2007 were found in the TJF region. The surface elevation difference over barren areas between the GLAS and DEM 1966 was 2.8 m±9.6 m (mean value ± standard deviation), and −0.67±9.3 m between the GLAS and SRTM. This is less than that found in Tien Shan Mountains [34]. The available number of ICEsat GLAS data limited the spatial correction of the DEM 1966 and SRTM. Thus, the corrections were made on the difference between the DEMs rather than the DEM itself.

**Figure 2:**
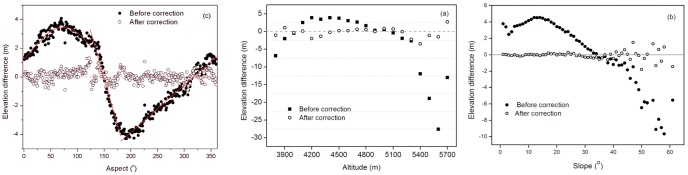
The before and after correction average surface elevation differences between the SRTM and the DEM 1966 over the barren terrain of the Tuanjiefeng region. (a) elevation zone, (b) slope, and (c) aspect.

Systemic biases can be found among different DEMs [29], [42]–[44]. These biases are a result of the combined effect of a co-registration shift [29], different resolution [45], and the C-band radar penetration of the SRTM signals [46]. A coarse resolution will underestimate elevation in a glacier area [45] (Figure 2a), and the co-registration shift leads to a clear sinusoidal relationship between the elevation difference and the aspect [29], [44].

Studies have shown that a sinusoidal relationship between the elevation difference and the aspect occurs across the entire aspect range [29], [44]. Thus, biases related to the aspect were corrected for the elevation difference between the SRTM and the DEM 1966. The co-registration shift between the SRTM DEM and the DEM 1966 varies from 10 to 60 m. This was assessed in detail by comparing 19 tie points extracted from the SRTM DEM and DEM 1966. This uneven error distribution probably attributed to the uneven distribution of the triangulation points. The transform points that are closer to the triangulation points will be more accurately transformed [47].

However, an ideal the sinusoidal relationship between the elevation difference and aspect is not ideal for the whole aspect range in the TJF region (Figure 2c). A clear sinusoidal relationship was found between 0 and 124 degrees, and two linear relationships identified between 125 and 181 degrees and between 182 and 360 degrees (Figure 2c). Therefore, three different models were applied to correct aspect bias.
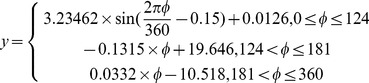
(5)where *φ* is the aspect in degree and *y* is the elevation difference.

After the aspect-related bias correction, the bias over the barren areas in the TJF region was −0.67±12.63 m. A nearly linear relationship was still observed between the elevation difference and slope after the aspect-related bias correction. This bias is probably due to different resolutions. It was corrected via the method [45], which used the relationship between the elevation differences and the maximum curvature of each pixel taken from the 30 resolution DEM with a 5×5 kernel size established on ice-free terrain. After the aspect-related and slope-related bias correction, the bias over the barren areas in the TJF region decreased to 0.06±8.73 m.

The relationship between the elevation difference between the SRTM and the DEM 1966 and the altitude (Figure 2a), slope (Figure 2b), and aspect (Figure 2c) over barren areas before and after the aspect-related and slope-related bias correction were compared. The relationship between the surface elevation difference and the altitude zones (Figure 2a) suggested that after the correction elevation difference was not altitude dependent. The relationship between the surface elevation and slope (Figure 2b) and the aspect (Figure 2c) before and after correction suggested that the correction significantly improved the accuracy of elevation difference calculation. The average surface elevation difference in glacier area is 8.34 m and 7.27 m before and after the correction, which means that a 12.8% error was corrected. Therefore, the ice surface elevation difference and ice volume change reported [27] were probably overestimated.

### Elevation change uncertainty

In general, the best way to estimate the absolute errors in elevation data is to use ground control points (GCPs) from a more accurate elevation data set [48], [49]. Due to the fact that no higher resolution elevation datasets were available, we used relative error estimated from residuals of the difference between the DEM 1966 and the SRTM DEM over stable barren terrain. Standard principles of error propagation were used for estimating the errors between the two DEMs [50]. Following the standard principles of error propagation, the mean standard error of the linear model is calculated using the following equation:
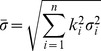
(6)where 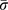 is the mean standard error, 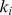 is the linear model slope, and 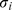 is the standard error of the regression model. Here, the parameter 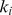 for 3 linear models is −0.1315, 0.0332, and 3.6036, and 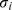 for the three linear models is 0.47, 0.32, and 0.11. Then:
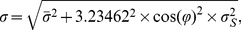
(7)where 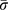 is the value obtained from Equation 6, 3.23462 is the regression parameter mentioned in Equation 6, and 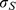 is the standard error of the sinusoidal model (i.e., 0.62). The minimum error was 0.59 m, and the maximum error was 2.09 m. When analyzing and quantifying glacier elevation changes, the spread of elevation changes should be considered, and the mean of the elevation changes over a particular area (e.g., a glacier or glacial zone) should also be considered. The standard error equation for the mean is calculated using the following equation:
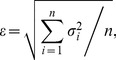
(8)where 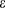 is the mean error of the measurement area, *n* is the total number of measurements, and 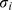 is standard error of the 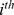 pixel obtained from Equation 8.

### Ice volume change and uncertainty

Ice volume change was calculated based on the glacier surface elevation change using the following three methods: 1) The arithmetic mean of the glacier elevation differences multiplied by the total glacier area, 2) An area-weighted average per 100 m of elevation zone, and 3) An area-weighted average for each glacier.

The change in ice volume was determined by changes in the glacier area and the ice surface elevation change as expressed in equation 8. The error can be estimated using Equation 9 according to the standard principles of error propagation. More specifically, the change in ice volume is expressed as follows:
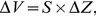
(9)where *S* is the total glacier area obtained from measurements, and 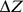 is the elevation change. The error is estimated using the following equation:
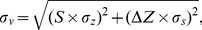
(10)where 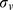 is the error for the ice volume change, 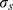 is the error for area, and 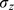 is the error for the elevation change.

## Results

### Changes in glacier area in the TJF region

The total glacier area in the TJF region was 162.70±3.07 km^2^ in 1966; interestingly, it decreased to 146.60±5.55 km^2^ by 2010. The overall decrease in the glacier area was 16.1±6.34 km^2^ (9.9±3.9%) from 1966 to 2010, accounting for 0.37 km^2^·a^−1^ (Table 2). The average annual glacier shrinkage was 0.15% a^−1^ from 1966 to 1995, 0.61% a^−1^ from 1995 to 2000, 0.20% a^−1^ from 2000 to 2006, and 0.45% a^−1^ from 2006 to 2010.

**Table 2:** Changes in the glacier areas from 1966 to 2010 in the Tuanjiefeng region, Qilian Mountain, China.

Year	Glacier area (km^2^)	Change in percentage from 1966 (%)	
		Total	Annual
2010	146.60±5.55	−9.9±3.9	−0.45
2006	149.31±5.51	−8.2±3.9	−0.20
2000	151.11±2.83	−7.1±2.6	−0.61
1995	155.84±5.68	−4.2±4.0	−0.15
1966	162.70±3.07	-	-

**Figure 3:**
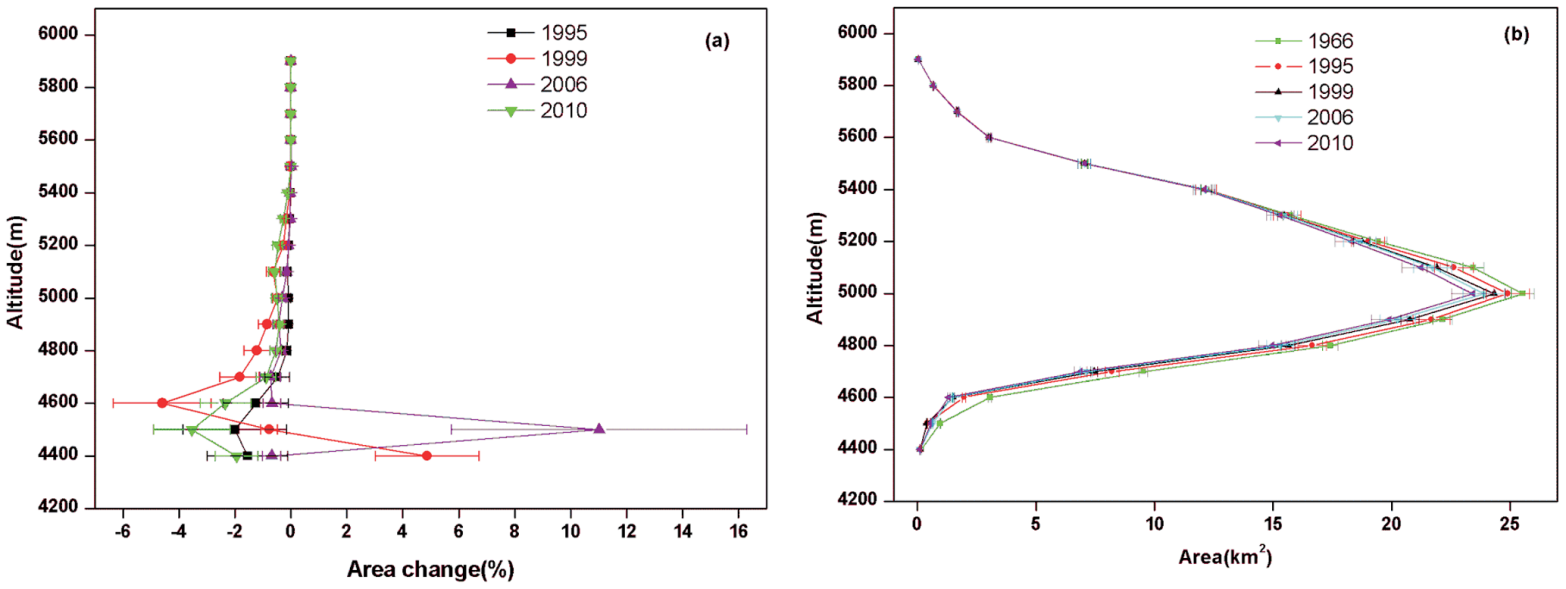
The average annual changes in glacier area relative to the area measured in 1966 along the altitude zone over the time periods of 1966–1995, 1995–1999, 1999–2006, and 2006–2010 in the Tuanjiefeng region. (a) is relative change(%), and (b) is absolute change (km^2^).

The relationship between glaciers' annual area change and elevation zone (Figure 3a) suggested that glacier's area change is much larger when the glacier is located below 4800 m ASL than above this line, and glacier's annual area change is the most pronounced around 4500 m ASL. In fact, the area of all glaciers above 4500 m ASL elevation shrunk during all study periods.

Also note that the annual area change of glacier is typically not consistent when comparing the periods 1995–1999, 1999–2006, and 2006–2010. For example, the annual area change of glaciers is about 11% a^−1^ in 4500 m elevation zone from 1999 to 2006, but it is about 4.8% a^−1^ during the period from 1995 to 1999 at the 4400 m elevation zone; this suggests that the glacier area even expanded during some periods. These results indicate that the relationship between the expansion and contraction of the glacier area and the elevation zone is complex.

The relationship between the glacier area and the elevation zone (Figure 3b) suggested that the maximum area change occurred around 5000 m ASL during 1966 to 2010, and the area of the glaciers located above 5500 m ASL are stable. The maximum absolute glacier area change occurred at 4800 m ASL from 1966 to 1995, and shifted to 5100 m ASL from 2006 to 2010. Note that the glacier area between 4400 m ASL and 4600 m ASL, i.e., near the glacier terminus, has been nearly a constant (i.e., above zero) since 1995.

### Changes in glacier area and length of larger glaciers

Four larger glaciers (i.e., greater than 5 km^2^), including 5Y445G0020, 5Y445G0026, 5Y445G0028, and 5Y445G0031, were selected for analysis of the changes in glacier area (Table 3) and length (Table 4) during the periods from 1966 to 1973, 1973 to 1995, 1995 to 1999, 1999 to 2006, and 2006 to 2010.

**Table 3:** Changes in areas of four typical glaciers (km^2^) around Tuanjiefeng region in 1966, 1973, 1995, 2000, 2006, and 2010, respectively.

Glacier ID	Year	Area(km^2^)	Change (%) relative to 1966	
			Total	Annual
5Y445G0020	2010	15.26±0.58	−6.27±4.04	−0.58
	2006	15.62±0.58	−4.05±4.04	0.67
	2000	15.02±0.28	−7.74±2.57	−0.46
	1995	15.37±0.56	−5.59±3.93	−0.23
	1973	16.18±1.31	−0.61±8.27	−0.09
	1966	16.28±0.31		
5Y445G0026	2010	5.07±0.19	−11.83±3.82	−1.83
	2006	5.47±0.20	−4.87±3.97	−0.64
	2000	5.69±0.11	−1.04±2.7	−0.35
	1995	5.79±0.21	0.7±4.12	−0.05
	1973	5.86±0.47	1.91±8.39	0.27
	1966	5.75±0.11		
5Y445G0028	2010	6.92±0.26	−16.43±5.31	−0.21
	2006	6.98±0.26	−15.7±5.31	−0.28
	2000	7.1±0.13	−14.25±3.59	−0.49
	1995	7.28±0.27	−12.08±5.49	−0.46
	1973	8.10±0.65	−2.17±11.64	−0.31
	1966	8.28±0.16		
5Y445G0031	2010	6.41±0.24	−5.48±4.75	0.08
	2006	6.39±0.12	−5.75±3.08	−0.33
	2000	6.52±0.24	−3.83±4.75	−0.36
	1995	6.64±0.24	−2.06±4.75	−0.09
	1973	6.78±0.55	0	0
	1966	6.78±0.13		

**Table 4:** Changes in lengths of four typical glaciers (m) around Tuanjiefeng region in 1966, 1973, 1995, 2000, 2006, and 2010, respectively.

Glacier ID	Year	Length (km)	Change (%) relative to 1966	
			Total	Annual
5Y445G0020	2010	6724.3±30	−2.82±0.48	0
	2006	6724.3±30	−2.82±0.48	1.88
	2000	6042.1±15	−12.68±0.31	−0.33
	1995	6143.6±30	−11.21±0.48	−0.27
	1973	6538.8±57	−5.5±0.85	−0.79
	1966	6919.2±15		
5Y445G0026	2010	5678.9±30	13.54±0.48	0.003
	2006	5678.1±30	13.52±0.48	−0.07
	2000	5703.1±15	14.02±0.31	0.16
	1995	5657.0±30	13.1±0.48	0.42
	1973	5177.9±57	3.52±0.85	0.5
	1966	5001.7±15		
5Y445G0028	2010	5011.5±30	−32.51±0.48	−0.97
	2006	5214.6±30	−29.78±0.48	−0.69
	2000	5441.0±15	−26.73±0.31	−0.96
	1995	5716.2±30	−23.02±0.48	−0.92
	1973	7176.1±57	−3.36±0.85	−0.48
	1966	7425.6±15		
5Y445G0031	2010	3858.1±30	−16.92±0.48	0.48
	2006	3785±30	−18.49±0.48	0
	2000	3785±15	−18.49±0.31	−0.78
	1995	3939.7±30	−15.16±0.85	−0.69
	1973	4643.6±57	0	0
	1966	4643.6±15		

The glacier area of 5Y445G0028 receded 1.36±0.30 km^2^ (i.e., −16.43±5.31%) from 1966 to 2010, which was greater than the average of the entire TJF region (i.e., −9.9%), and the recession rates have been greater than 0.40% a^−1^ from 1973 to 1995 and 1995 to 2000, (Table 3). The largest recession rate was 1.83% a^−1^ for 5Y445G0028 from 2006 to 2010. The largest glacier area expansion speed is 0.67% a^−1^ for 5Y445G0020 from 2000 to 2006.

Great differences in the glacier area change were observed during the same time periods. For example, the glacier area changes varied from −0.64% a^−1^ for 5Y445G0026 to 0.67% a^−1^ for 5Y445G0026 during 2000 to 2006 (Table 3), and the average for the whole TJF region was −0.20% a^−1^ (Table 2). This indicates that the change in glacier area is not only affected by climate change but also other factors, such as glacier size, slope, aspect, etc. It also indicates that great uncertainty probably exists when presenting some “benchmark” glacier area changes as representative for regional glacier area changes.

Glacier length fluctuations are not always in accordance with that of glacier area. For 5Y445G0020, the glacier area contracted or expanded at the rate of −0.09, −0.23, −0.46, 0.67, and −0.58% a^−1^ while the glacier length retreated or advanced at the rate of −0.79, −0.27,−0.33, 1.88, and 0% a^−1^ during the periods of 1966 to 1973, 1973 to 1995, 1995 to 2000, 2000 to 2006, and 2006 to 2010, respectively. However, for 5Y445G0026, the glacier area contracted by 0.35% a^−1^ during 1995 to 2000, but the glacier length advanced by 0.16% a^−1^ during the same time period (Table 4).

Great variations in glacier length changes were also observed within the same period. For example, the glacier length change was −0.69% a^−1^ for 5Y445G0028, and 1.88% a^−1^ for 5Y445G0020 during the period from 2000 to 2006 (Table 4). This evidence also indicates that glacier length change is not only affected by climate change but also other factors.

### Changes in glacier elevation from 1966 to 1999

**Figure 4:**
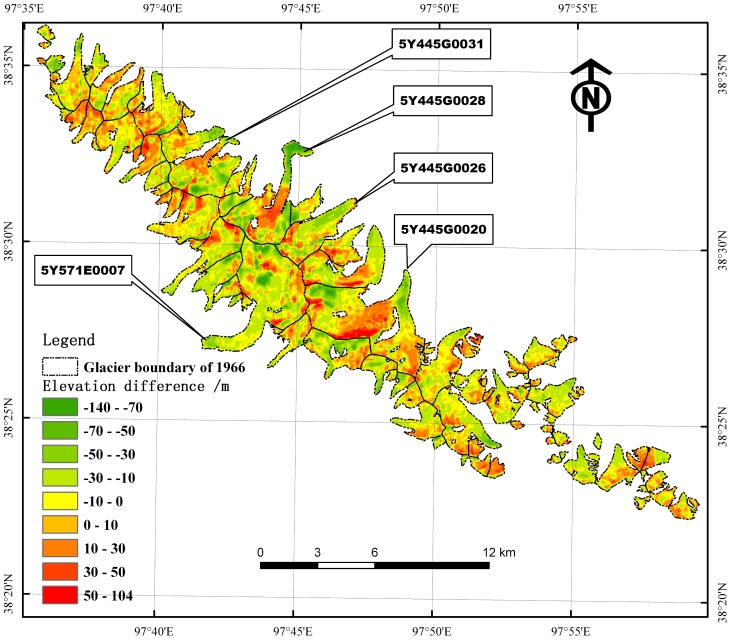
Spatial distribution of the difference in glacier surface elevation between the corrected SRTM data and the DEM 1966 of the Tuanjiefeng region.

The mean glacier surface elevation change for the entire TJF region was −7.3±1.5 m from 1966 to 1999 (Figure 4), thereby indicating an annual change of 0.21±0.04 m·a^−1^. The surface elevation for about 65% of grid area decreased, i.e., mostly in the glacier ablation zone and in areas of accumulation on mountain crests. We also observed extreme thinning (i.e., locally exceeding 90 m) at the low-lying terminus of glacier 5Y445G0028, i.e., the lowest point in the TJF site, which is believed to be to the result of melting of ice. Extreme thinning was also observed on the steep ridges of glacier 5Y571E0007 (Figure 4), which was probably caused by an avalanche. Glacier thickening occurred mostly in firn areas.

Note that some measurable mass displacements were observed down the glacier, possibly as a result of sliding, avalanches, or other forces, all of which can change the ice thickness distribution but not the ice volume loss or gain. For example, the terminus of glacier 5Y445G0026 had advanced 14.02% (Table 4), while the glacier area of glacier 5Y445G0026 contracted by 1.04% during the period from 1966 to 2000 (Table 3), and the average ice thickness change −7.9±1.5 m from 1966 to 1999.

Glaciers in the study area are separated into northeasterly and southwesterly aspects by the main ridgeline (Figure 1), and we found a regional difference in the glacier surface elevation changes. Glaciers with a northeastern aspect thinned by 8.3±1.4 m, i.e., almost twice as much as those with a southwestern aspect, which thinned by 4.3±1.3 m. Interestingly, 76% of the glaciers with ice thickening have a northeasterly aspect. However, the area change for glaciers with a northeasterly aspects is only a little larger than that of the glaciers with the southwesterly aspects (i.e., −7.5% and −6.4%, respectively). This agrees with results from previous research [51], which reflect the climatic conditions in the Qilian Mountains where the annual precipitation and temperature decreases from northeast towards the southwest.

### Ice volume changes from 1966 to 1999

As determined by method 1 for the measurement of ice volume changes as described in the methods section, the change in ice volume for the entire study area was about −11.88±2.45×10^8^ m^3^. For method 2 and method 3, the calculated change was about −11.71±2.37×10^8^ and −11.56±2.32×10^8^ m^3^, respectively. The difference between the results of the three methods was less than 1.5%. A previous study [23] found the difference between an area-weighted average per 100 m of elevation zone and the arithmetic mean of ice thickness was only 1.7%. The results from method 2 showed that the maximum change occurred at 5200 to 5300 m ASL, which include the mass migration area shown in Figure 4.

Mass loss still occurs above 5500 m ASL as a result of glacier surface elevation changes without area change. This change is represents real change and is easily underestimated by SRTM DEM due to the low resolution with these images. Similar findings were described by previous study [52], and other explanations, including heterogeneous radar penetration and instrumental biases, have been suggested [42].

**Figure 5:**
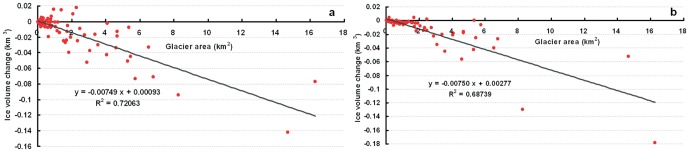
Relationship between changes in ice volume for each glacier and glacier size. (a) ice volume calculated from the glacier surface elevation change between the corrected SRTM and DEM 1966, and (b) ice volume derived from the statistical relationship between the glacier area and ice volume in the Tuanjiefeng region.

The relationship between ice volumes changes for each glacier and the glacier area (Figure 5a) suggests that the decrease in ice volume is nearly linear with glacier area, and the negative correlation is significant (i.e.,α = 0.01). Therefore, large glaciers experienced the greatest mass loss with no dependence on geographic location assuming that they are located in the same climate zone. The same phenomenon has been also reported by Paul and Haeberli [19] for glaciers in Swiss Alps.

A Previous study [26] found a strong statistical relationship between the area and volume of a single glacier in western China, which is expressed as follows:
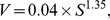
(11)where *S* is area (km^2^) and *V* is volume (km^3^). We used glacier area in 1966 and 1999 to calculate ice volume change during the period from 1966 to 1999. The relationship between ice volume change and glacier area (Figure 5b) suggests that the decrease in ice volume change is nearly linear with glacier area, and the relationship is significant (i.e.,α = 0.01). The slope in Figure 5b is nearly the same with that in Figure 5a (i.e., −0.00750 and −0.00749, respectively), thereby indicating that the ice volume change difference between estimated by the two methods is not strongly correlated with glacier area. Thus, the error for the total ice volume as estimated by the glacier area-volume relationship can be approximated as the differences between the two interceptions (0.00184 km^3^). The total ice volume change was −9.53±2.54×10^8^ m^3^ in the TJF region during the period from 1966 to 1999, which is 17.4±4.6% less than the ice volume change estimated by the glacier surface elevation change. This indicates that the ice volume probably underestimated when using Equation 11.

## Discussion

### Glacier advances or surges

Glacier surge are short-lived events where a glacier can advance substantially, moving at velocities up to 100 times faster than normal. In some glaciers, surges can occur in fairly regular cycles and may include 15 to 100 or more surge events per year. In other glaciers, surging is unpredictable [53]. A previous study found that mass displacement to down-glacier is an important signal that occurs before a glacier surge [54]. Such mass displacements were found in three glaciers, i.e., 5Y445G0020, 5Y445G0028, and 5Y445G0031 during the period from 1966 to 1999. 5Y445G0020 advanced from 1999 to 2006, and 5Y445G0031 and 5Y445G0020 advanced from 2006 to 2010, thereby indicating that the prediction of the actual surge time is still difficult to even mass displacement downward was found.

This kind of mass displacement was also found in other Asian glaciers, and these observations were more frequently noted after 1999 than before [28], [37]. They were all assumed to potentially surging glaciers. Surging glaciers also were also found in the Tibet plateau based on glacier surface velocities measurement [55]. Sudden glacier surges are difficult to predict via direct monitor glacier area/length changes.

Most surge glaciers, including our study glaciers, retreated due to the climate warming during the period from 1980 to 2000 before they actually surged or advanced [6]. A previous study [28] suggested that surging glaciers in Karakoram showed slight mass gain. Another previous study [37] assumed surging glaciers correspond with mass gain, but this cannot be confirmed due to a lack of upper part elevation change data. Furthermore, we must consider whether the climate warming from 1980 to 2000 accelerated glacier advanced. Among the four typical glaciers in the TJF region, one glacier advanced from 1999 to 2006, whereas two glaciers advanced from 2006 to 2010. More glaciers seemingly exhibited advances after the increased climate warming than before. However, the number of studied glaciers is rather small, thereby preventing any type of generalizability of the results. However, this phenomenon was also found in Tien Shan [37], but the mechanism for this phenomenon is still unclear.

### Glaciers response to climate change

Climate change, especially temperature change, is the fundamental factor underlying changes observed in glacier [56], [57]. Thus, meteorological data obtained from the Tuole weather station (38.80°N, 98.42°E; 3367 m ASL), which is located about 6.7 km northeast of the TJF, were used to analyze the driver of glacier change in the TJF region. The Tuole station reported a clear increasing trend by 0.023°C a^−1^ in the average summer temperature from 1957 to 2003, whereas the annual precipitation varied significantly and showed a slightly increasing trend of 0.87 mm·a^−1^ during the same period [27]. The increase in the summer average temperature will lead to an increase in the annual positive degree-day. The increase in the annual positive degree-day when the summer air temperature will result in more glacier ablation and runoff and greater glacial mass balance sensitivity to temperature [58].

**Figure 6:**
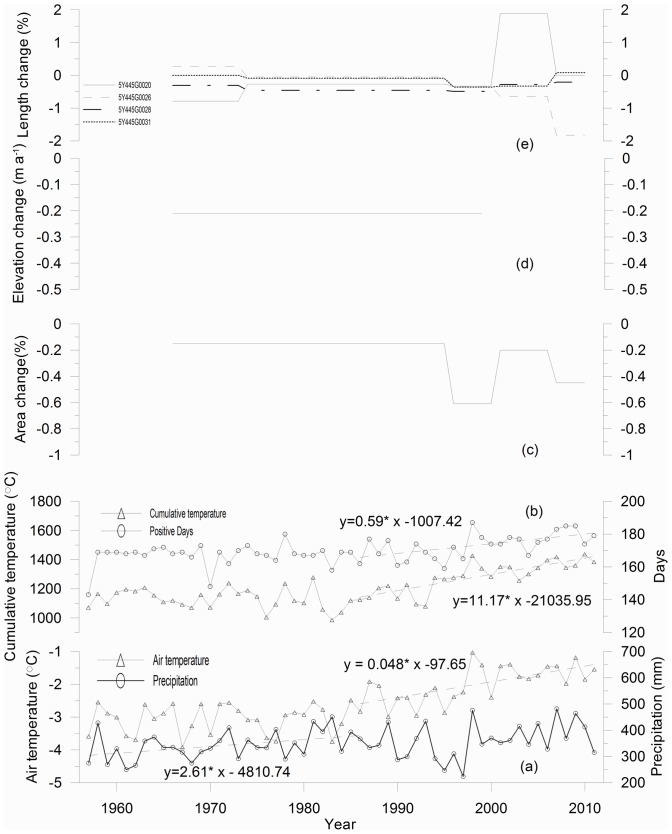
Comparison of changes in precipitation and temperature and glacier surface elevation and length. (a) annual precipitation (mm) and annual temperature (°C), (b) positive cumulative temperature (°C) and positive days at the Tuole meteorological station, (c) average annual glacier area change (% a^−1^), (d) average annual glacier surface elevation (m a^−1^), and (e) average annual glacier length of typical larger glaciers (i.e., 5Y44G0020, 5Y44G0026, 5Y44G0028, and 5Y44G0031) from 1957 to 2011 in the Tuanjiefeng region. All the linear fits are at significant (i.e., p<0.05).

A previous study suggested that the climate of northwest China had shifted from warm and dry to warm and wet since 1987 [59]. Detection of transition points in annual air temperature at the Tuole station via the Mann-Kendall method indicated that the tipping point occurred in 1986 [60]. Thus, the time series of annual precipitation, average air temperature, positive cumulative temperature, and positive days during which daily air temperature was equal to or more than 0°C during 1957 to 2011 were divided into two periods (i.e., before and after 1987) for analysis (Figure 6).

A slight increase in precipitation of 2.61 mm·a^−1^ (i.e., p<0.05) from 1957 to 1986 was observed, while the annual average temperature was showed no significant trend (Figure 6a); moreover, the positive cumulative temperature and positive days also showed no significant trend (Figure 6b). These conditions would have contributed to higher accumulation and the corresponding positive glacier mass balance, which may perhaps lead to some glacial advance. For example, the annual glacier area was only −0.15% a^−1^ during the period from 1966 to 1995 (Figure 6c), but the glacier 5Y445G0026 advanced during this period (Figure 6d).

Precipitation was nearly stable, and a rapid significant (i.e., p<0.001) increase in annual average temperature by 0.048°C a^−1^ (Figure 6a) was observed during 1987 to 2011; moreover, a significant increase in positive days by 0.59 day a^−1^ and a significant (i.e. p<0.001) increase in positive cumulative temperature by 11.17°C a^−1^ (Figure 6b) was observed during the same period. These conditions would contribute to intense ablation and corresponding negative glacier mass balance, which would lead to accelerated glacier area shrinkage and glacier thinning. The annual glacier area change reached −0.61% a^−1^ during 1995 to 2000 (Figure 6c).

On the other hand, the responses by individual glaciers to climate change varied greatly, which is probably due to different response times. For example, the area of glacier 5Y445G0020 expanded by 0.67% a^−1^ from 2000 to 2006, while the area of glaciers 5Y445G0026, 5Y445G0028, and 5Y445G0031 contracted by 0.64, 0.28, and 0.33% a^−1^, respectively (Table 3). The length of glacier 5Y445G0020 advanced by 1.88% a^−1^ from 2000 to 2006, while the length of glaciers 5Y445G0026, 5Y445G0028, and 5Y445G0031 retreated by 0.07, 0.69, and 0.00% a^−1^, respectively (Figure 6e). This indicates that great uncertainties are likely still present when using the observed mass balance of a typical glacier to represent regional glacial changes.

### Glacier response time and implications to glacier area change simulation

A previous study [61] suggested that glacier surface elevation change can depend not only on current but also multi-decadal average accumulation rate. Another study [58] implied that the area change of a glacier probably has a time lag (i.e., about 10 years) when compared to the mass balance. The response time depends on the glacier size and ice thickness and the slope of the glacier surface [62]. The varied glacier response time leads to more uncertainty when modeling the glacier area changes.

Recently, some models [12], [58], [63], [64] have developed schemes for simulating glacial area changes using annual mass balance; but they considered all single glaciers in one grid box or sub-basin as one single glacier, and they assumed that the expansion or recession of a glacier occurred at the lowest glacier elevation zone. However, this is not always the case when we consider all glaciers as one glacier due to glacier dynamics. For example, in our study, the most pronounced glacier area contraction occurred around 5000 m ASL from 1966 to 2010 (Figure 3b), while the glacier terminus elevation ranged from 4400 to 4600 m ASL in the TJF region. Intense ice thinning was also observed around 5000 m ASL. These results indicate that the glacier area change and ice thickness change was not only caused by climate change, but also glacier dynamics, which are affected by local factors (e.g., slope, aspects, and glacier size). Glacier area change schemes should consider different responses to climate change by various individual glaciers in each grid box or sub-basin, especially when the resolution of the model is relative large or includes many individual glaciers.

## Conclusions

Topographic maps, multi-temporal remote-sensing images, and SRTM DEM were used to monitor changes in glacier area and ice surface elevation/volume from 1966 to 2010 in the TJF region. The following conclusions can be reached:

1. The overall change in the glacier area in the TJF region is −16.1±6.34 km^2^ (−9.9±3.9%) from 1966 to 2010, which translates to an average annual change of −0.37 km^2^·a^−1^. The average annual glacier change was −0.15% a^−1^ from 1966 to 1995, −0.61% a^−1^ from 1995 to 2000, −0.20% a^−1^ from 2000 to 2006, and −0.45% a^−1^ from 2006 to 2010. The annual area change for the studied glaciers was the most pronounced around 4500 m ASL. Glacier length and glacier area change varied greatly among different individual glaciers.
2. The mean glacier surface elevation change for the entire TJF region was −7.3±1.5 m from 1966 to 1999, which suggests an annual change of −0.21±0.04 m·a^−1^. The surface elevation for about 65% of the grid area decreased, mostly in the glacier ablation zone and in areas of accumulation on mountain crests.
3. Glaciers with a northeastern aspect thinned almost twice as much as those with a southwestern aspect. However, the area change for glaciers with northeasterly aspects was only a little larger than that of glaciers with southwesterly aspects.
4. The change in ice volume as estimated from ice surface elevation changes for the TJF region was about −11.88±2.45×10^8^ m^3^, which is about 17.4% larger than that estimated by ice volume-area relationship.
